# Metal–Phenolic Network-Directed Coating of *Lactobacillus plantarum*: A Promising Strategy to Increase Stability

**DOI:** 10.3390/foods14132277

**Published:** 2025-06-26

**Authors:** Haoxuan Zhang, Huange Zhang, Hao Zhong

**Affiliations:** College of Food Science and Technology, Zhejiang University of Technology, Hangzhou 310014, China

**Keywords:** *Lactobacillus plantarum* YJ7, metal–phenol networks, single-cell encapsulation, stability

## Abstract

*Lactobacillus plantarum* exhibits probiotic effects, including regulating the balance of the intestinal microbiota and enhancing immune function. However, this strain often experiences viability loss upon ingestion due to harsh conditions within the human digestive tract. This study aimed to evaluate the efficacy of metal–phenol networks (MPNs) fabricated via three polyphenols—tannic acid (TA), tea polyphenol (TP), and anthocyanin (ACN)—combined with Fe(III) coatings in protecting *Lactobacillus plantarum* during simulated digestion and storage. The results demonstrated that MPNs formed a protective film on the bacterial surface. While TA and ACN inhibited the growth of *Lactobacillus plantarum* YJ7, TP stimulated proliferation. Within the MPNs system, only Fe(III)-TA exhibited growth-inhibitory effects. Notably, ACN displayed the highest proliferation rate during the initial 2 h, followed by TP between 3 and 4 h. All MPN-coated groups maintained high bacterial viability at 25 °C and −20 °C, with TP-coated bacteria showing the highest viable cell count, followed by TA and ACN. In vitro digestion experiments further revealed that the Fe(III)-ACN group exhibited the strongest resistance to artificial gastric juice. In conclusion, tea polyphenol and anthocyanin demonstrate superior potential for probiotic encapsulation, offering both protective stability during digestion and enhanced viability under storage conditions.

## 1. Introduction

Probiotics are defined as “living microorganisms that, when given in sufficient amounts, produce health benefits to the host” [1,2]. A large number of clinical trials have found that probiotics are beneficial to human health, disease prevention, and treatment [3,4,5]. *Lactobacillus plantarum* is one of the most widely used probiotics, which has great beneficial effects on human and animal health, and it is widely distributed in fermented food, meat, and the human gastrointestinal tract [6,7]. Studies have shown that *L. plantarum* can secrete lactic acid and various metabolites during the colonization process, which can effectively inhibit the growth of pathogenic bacteria, regulate immune function, restore intestinal homeostasis, and enhance intestinal barrier function [8,9]. Among them, its metabolite short-chain fatty acids can provide energy, and they also play a role in gene regulation and the differentiation and expansion of T cell subsets [10]. In addition, several studies have shown that different strains of *L. plantarum* can alleviate health problems such as inflammatory bowel disease [11,12], diabetes [13,14], hyperuricemia [15], osteoporosis [16], and memory disorder [17] by regulating the composition of the gut microbiota.

In general, to ensure that probiotics can exert their health benefits, the amount of probiotics taken orally and surviving to reach the colon should be greater than 1 × 10^7^ cfu/mL [1]. However, probiotics are inactivated by various factors during processing, storage, transportation, sale, and consumption, which limits the beneficial effects of probiotics [18]. For example, after being ingested by the human body, probiotics are inactivated by the extreme pH of the gastrointestinal tract or by bile salts that disrupt the integrity of their cell membranes. Therefore, it is necessary to develop a suitable embedding system to improve the stability of probiotics.

In recent years, single-cell encapsulation technology, as a coating technology, has gradually attracted wide attention because of its ability to explore cell heterogeneity at the individual level and its characteristics of protection, small size, and strong versatility [19,20]. It can create artificial shells around probiotics to improve their resistance to physical and chemical stress [21]. Among them, the single-cell delivery vector constructed by the metal–phenol network (MPN) has attracted much attention. Since iron ions have less biotoxicity than other metal ions, protons of phenolic compounds can be replaced at low pH, and deprotonated phenolic groups act as good ligands for metal cations, the MPN has been widely used to construct multifunctional surfaces and as an adhesive layer for probiotics and protective wall materials to enhance the protective ability against probiotics [22,23]. In addition, due to the antioxidant activity, pH response, and antibacterial properties of polyphenols, the cell coating formed after the chelation of polyphenols with metal ions to form the MPN also has good antibacterial properties. For instance, the MPN was employed to embed Saccharomyces cerevisiae for enhanced UV protection [24]; the MPN protected *Escherichia coli* Nissle 1917 against antibiotics [25]; and *E. Coli* was embedded within the MPN to withstand harsh conditions such as oxygen exposure and freezing [26].

However, the MPN is less applied in the oral encapsulation of probiotics, but many studies have shown that the MPN nanocoating has the ability to protect probiotics. For instance, the MPN formed by the combination of TA and Fe(III) has been proven to protect anaerobic bacteria from various environmental stresses, including oxygen environments and freeze-drying [26]. The nanocoating composed of TA and Fe(III) can protect probiotics from various antibiotics [25]. The strong interaction between TA and mucin has been proven to significantly improve the mucosal adhesion of probiotics in the intestine [27].

Fe(III) is low in cost, recognized as safe (GRAS) by the US Food and Drug Administration (USFDA), and has low toxicity. TA, ACN, and TP are all natural polyphenols widely present in plants, which are recognized for their safety. Moreover, the hydroxyl group of TA is connected to multiple galloyl groups, and each galloyl group contains three adjacent phenolic hydroxyl groups. TP is mostly a polyphenol containing more than two adjacent hydroxyl groups, with multiple highly active coordination sites, and it has inhibitory activity against a variety of bacteria. The ACN molecule contains multiple phenolic hydroxyl groups. These abundant ortho phenolic hydroxyl structures ensure that they can rapidly and efficiently form a dense network structure with Fe(III), evenly coating the surface of probiotics. These abundant ortho-phenolic hydroxyl structures ensure that they can rapidly and effectively form stable octahedral complexes with Fe (III), and the high charge(III) of Fe(III) allows for the formation of multitooth coordination structures with polyphenols, which evenly coat the surface of probiotics. We compared the survival performance of single-cell carriers prepared with different polyphenols to identify a more suitable encapsulation material for *Lactobacillus plantarum*. This aimed to enhance the stability of *Lactobacillus plantarum* and further improve the utilization rate of this probiotic.

## 2. Materials and Methods

### 2.1. Chemicals and Reagents

The *Lactobacillus plantarum* YJ7 samples used in this study were all from laboratory storage. Tea polyphenols, anthocyanins, and tannins were purchased from Shanghai Yuanye Biotechnology Co., Ltd. (Shanghai, China). Ferric chloride was purchased by Shanghai Aladdin Biochemical Technology Co., Ltd. (Shanghai, China). The CCK-8 kit is produced by Beijing Solaibao Technology Co., Ltd. (Beijing, China). Tris-HCl powder (pH 7.4) was purchased from Shanghai Aladdin Biochemical Technology Co., Ltd. (Shanghai, China). MRS Broth and AGAR are produced by Hangzhou Best Biotechnology Co., Ltd. (Hangzhou, China). Rhodamine B (analytical pure) was purchased from Shanghai Maclin Biochemical Technology Co., Ltd. (Shanghai, China).

### 2.2. Preparation of Single-Cell Encapsulation Carriers

To prepare single-cell nano-encapsulated carriers, Lactobacillus plant-based YJ7 was first cultured in an incubator at 37 °C for 20 h and then subcultured 2–3 times to enhance the viability of the strain. The preparation steps are as follows: Take 1 mL of the culture medium, centrifuge it (1760× *g*, 4 °C, 4 min), wash it 1–2 times with 3 mL of normal saline to remove the excess medium, and resuspend it in 1 mL of Tris-HCl buffer to obtain a probiotic suspension with an activity of 10^8^ CFU/mL. Then, add 3 mL of tannic acid, tea polyphenols, and anthocyanin solutions (1 mg/mL) to the centrifuge tubes and mix them evenly on the vortex. Finally, add 1 mL of ferric chloride solution (1 mg/mL). After 30 min of reaction, centrifuge (1760× *g*, 4 °C, 4 min) and wash twice with normal saline. Resuspend in 5 mL of PBS buffer to obtain experimental group samples with different MPNs. In the control group, 1 mL of the bacterial liquid was centrifuged and washed twice (1760× *g*, 4 °C, 4 min), and then 5 mL of Tris-HCl buffer was added.

### 2.3. Characterization of Single-Cell Encapsulation Carriers

The morphology of the single-cell encapsulated carrier was observed by a transmission electron microscope (TEM) [28]. An appropriate amount of the sample solution was dripped onto the copper mesh, the sample was washed with the buffer (2 times, 5 min), and then the sample was completely dried. Then, Rhodamine B was mixed with tannic acid, tea polyphenols, and anthocyanins, the polyphenols labeled by Rhodamine B were left for 30 min, and the samples were prepared according to the above steps. The encapsulation of Lactobacillus plantarum by the coating was observed by confocal laser scanning microscopy (CLSM) [29]. In addition, the sample was resuspended in 5 mL of Tris-HCl buffer, 10 μL of the sample solution was placed in the electrode potential cell, and the particle size distribution and zeta potential of the samples were measured by dynamic light scattering (DLS).

### 2.4. Influence of Polyphenols on the Growth of Lactobacillus plantarum

The samples were prepared following the method outlined in 2.2. The gradient was diluted to OD_600_ = 0.5 in MRS, inoculated into 96-well plates, and incubated in a 37 °C incubator. The absorbance was measured at 600 nm every 1 h, and the growth curve was drawn to observe the effect of polyphenols on the growth of the bacteria. In addition, the prepared carrier sample was diluted to about 0.5 absorbance at 600 nm and cultured in an incubator at 37 °C. The absorbance was measured every 1 h, and its growth curve was drawn to observe the growth of the single-cell delivery carrier.

The bacterial viability was detected by cck-8, referring to the method of Yang Xinyuan, with slight modifications [30]. The cell viability was examined by the cell counting kit-8 (CCK-8). *Lactobacillus plantarum*, the initial product, and the final product were gradient-diluted in the culture medium to make OD_600_ = 0.5. A total of 190 μL of each of the three samples was inoculated into 96-well plates and cultured at 37 °C without shaking. In total, 10 μL of CCK-8 solution was added to each well. The OD values of the samples were recorded at 450 nm at 1 h intervals by a microplate reader.

### 2.5. In Vitro Storage and Gastric Digestion Experiments

In this study, the characteristics of the single-cell delivery vector were investigated by storage and gastric digestion experiments in vitro. Single-cell vector samples were prepared according to the method outlined in 2.2. Equal amounts of Lactobacillus plantarum samples and Fe(III)-TA, Fe(III)-TP, and Fe(III)-ACN samples (10^8^ CFU/mL) were evenly divided into three parts and placed at −20 °C, 4 °C, and 25 °C, respectively. After one week of storage, 100 μL of the solution was taken out, washed 2 times with sterile normal saline, the bacterial suspension was spread on MRS AGAR plates, incubated at 37 °C for 48 h, and then colony counting was performed to observe its storage stability.

Secondly, in order to further verify the improved stability of the MPN-based single-cell delivery vector, artificial gastric juices (pH 1.5, composed of 16.4 mL of 10% dilute hydrochloric acid, 1 g of pepsin, and 100 mL of water) were used to simulate the gastrointestinal environment and perform colony counting. We followed the steps outlined here: Take 1 mL of *Lactobacillus plantarum* YJ7 culture medium in logarithmic growth stage, wash it 2 times, centrifuge (1760× *g*, 4 °C, 4 min), and discard the supernatant, then resuspend it in 1 mL of Tris-HCl buffer, add different polyphenols, prepare a single-cell delivery carrier according to the above steps, and wash it with normal saline 1–2 times, and then discard the supernatant. A total of 3 mL of artificial gastric juice was added to the sample, which was digested for 1 h and cultured in an incubator at 37 °C for 48 h. Colony counting was performed to observe the growth of the single-cell delivery carrier.

### 2.6. Statistical Analysis

The data were expressed as mean ± SD. The *t*-test or one-way analysis of variance were used to evaluate the significance of the differences between the two groups and multiple groups, and statistical analysis was performed by Origin 2025 and IBM SPSS Statistics 18.0. *p* < 0.05 was considered statistically significant (* *p* < 0.05, ** *p* < 0.01, and *** *p* < 0.001).

## 3. Results and Discussion

### 3.1. TEM

**Figure 1:**
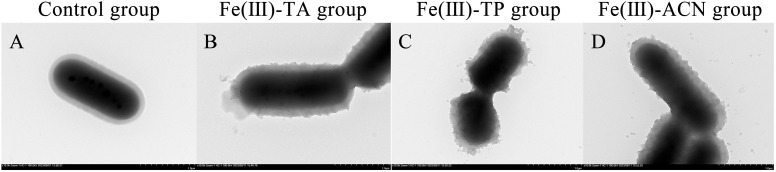
Electron micrographs of single-cell delivery carriers in three MPN systems under TEM. (**A**) Control group; (**B**) Fe(III)-TA group, (**C**) Fe(III)-TP group, and (**D**) Fe(III)-ACN group.

In this study, transmission electron microscopy (TEM) was used to observe the morphology of single-cell embedding carrier samples constructed with different phenols (Figure 1). According to the electron microscope image, the unembedded bacterium was an oval vesicle with a smooth surface (Figure 1A). After the MPN was induced by different polyphenols and ferric chloride reagents, the surface of *Lactobacillus plantarum* was rough, and adhesion between the bacteria occurred (Figure 1B–D). This result is similar to the study of Yang Xinyuan [30], which may be due to the fact that a large number of catechol groups in polyphenols are replaced with amino groups on the surface of cell membranes and coupled with iron ions, aggregating on the cell surface to form a protective film, thus making the cell surface rough. At the same time, catechol groups are coupled with iron ions of another bacterial coating, which enhances the adhesion of the coating. It is more likely that multiple bacteria will cluster together [31]. Wang proposed that metal ions would affect the thickness and roughness of the MPN structure. compared to metals with different oxidation numbers (Cu^2+^/TA, Al^3+^/TA, and Zr^4+^/TA), the film thickness of Zr^4+^/TA was the thickest and Cu^2+^/TA capsules the thinnest [32]. Zhao et al.’s research also pointed out that after MPN encapsulation, the smooth surface of the bacteria also became rough [33]. This indicates that after MPN treatment, metal–phenol was adsorbed on the surface of the naked bacteria to form a coating.

### 3.2. CLSM

**Figure 2:**
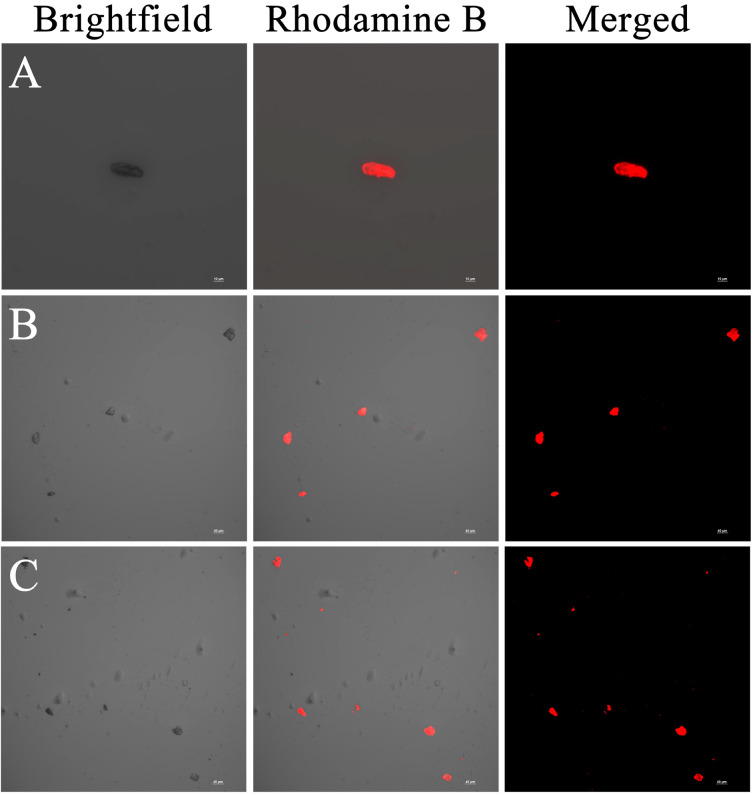
CLSM electron micrographs of single-cell delivery carriers in three MPN systems. (**A**) Fe(III)-TA group, (**B**) Fe(III)-TP group, and (**C**) Fe(III)-ACN group.

In order to further prove that the MPN system formed by polyphenols and Fe (III) can form film on the surface of *Lactobacillus plantarum* and confirm its film formation, TA, TP, and ACN were labeled with Rhodamine B solution, and the samples were prepared and observed under a confocal laser scanning microscope (Figure 2). The stabilization process of Fe (III) in the polyphenol network occurs through the formation of cross-linked ligand bonds, resulting in the formation of an MPN coating, which is then adsorbed onto the surface of bare bacteria. In the three groups of samples, the red light displayed by Rhodamine was in the same position as the bacteria in the samples, indicating that Rhodamine B successfully modified the polyphenols, and the labeled polyphenols were successfully coupled with iron and formed a layer of MPN nanomembrane on the surface of the bacteria. This phenomenon is similar to the CLSM observed by Le Ma et al. [34], which further proves that this experimental method can successfully prepare the required single-cell delivery vector.

### 3.3. Particle Size and Zeta Potential

**Figure 3:**
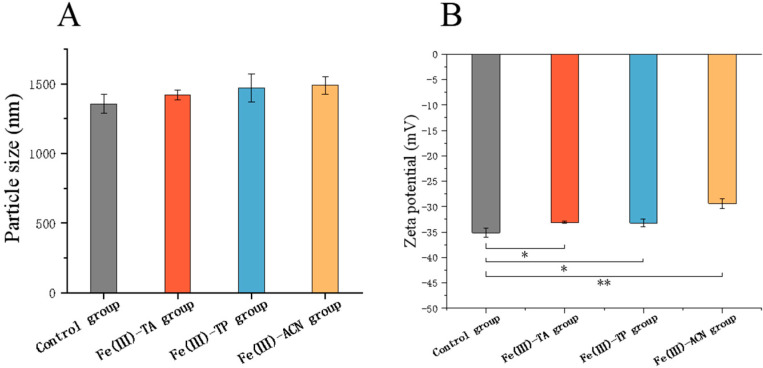
Determination of particle size and zeta potential of single-cell delivery carriers in three MPN systems. (**A**) Particle size, (**B**) zeta potential. * *p* < 0.05, ** *p* < 0.01.

In addition, we evaluated the characteristics of single-cell delivery vectors, including particle size and zeta potential (Figure 3). Dynamic light scattering (DLS) showed that the zeta potential of the control group was −35.14 ± 0.88 mV and the particle size was 1356.67 ± 67.66 nm. The particle sizes of the Fe(III)-TA group, Fe(III)-TP group, and Fe(III)-ACN group were 1421.00 ± 36.51 nm, 1471.33 ± 100.22 nm, and 1490.67 ± 63.12 nm, respectively. Although the particle size of the MPN groups was not significantly different from that of the control group, the increase in particle size indicated that the MPNs’ coating was successfully adsorbed on the surface of the bare bacteria. This might be because the bacteria surface was covered with a relatively thin carrier layer, meaning that the particle size difference was not obvious.

Zeta potential is an important characteristic characterizing the dispersion system (emulsions, liposomes, nanoparticles, etc.). Nanoparticles with a zeta potential greater than ± 30 mV are regarded as strong cations and strong anions. Generally speaking, nanoparticles with a higher zeta potential have a stronger electrostatic repulsive force, which helps to stabilize them and prevent their aggregation [35]. The potential of the Fe(III)-TA group was −33.09 ± 0.20 mV, and that of the Fe(III)-TP group was −29.39 ± 0.95 mV. The potential of the polyphenol group was significantly different from that of the control group, indicating that the MPN was successfully coated on the bacteria, but the coating thickness was thin. Among them, the stability of the tannic acid and tea polyphenol group was not as stable as the control group, but it was more stable than that of the anthocyanin group. Using TA as a model polyphenol, all of the selected metal ions (Cu^2+^, Fe^3+^, Al^3+^, Zr^4+^, and Ti4+) formed MPNs featuring zeta potentials ranging from −30 to −50 mV [32]. Then, using Fe(III) as the metal ion of the MPN and smaller phenolic ligands (TA, TP, ACN) will lead to the formation of an MPN with a more neutral zeta potential [36], thus increasing the bacterial potential after encapsulation. Zhang used the Fe(III)-TA system to enclose the bacterial species *S. aureus* [37], and the vector constructed by Fang’s TA-Ca^2+^ system had a significantly higher potential than that of the control group [38], which showed significant differences from the present experiment. The different changes in its potential may be due to the increase in the surface potential of bacteria caused by the binding of iron ion itself to specific sites of bacteria, and the impact of this reaction is more significant than that of the potential change caused by the MPN [39].

### 3.4. Effects of Single-Cell Encapsulation on the Growth of Lactobacillus plantarum

**Figure 4:**
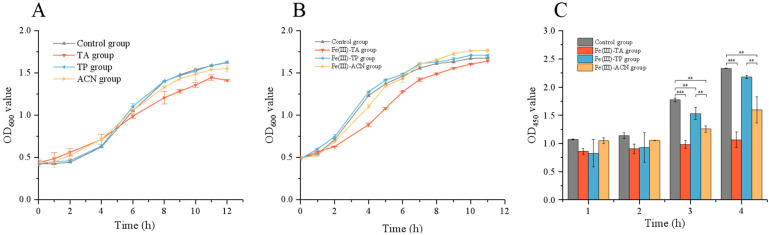
The effects of single-cell encapsulation on the growth of *Lactobacillus plantarum*. (**A**) Effects of three polyphenol materials on the growth curve of *Lactobacillus plantarum* YJ7, (**B**) the growth curve of *Lactobacillus plantarum* YJ7 in the encapsulated single-cell carriers, and (**C**) proliferation activity of single-cell delivery carriers in three MPN systems determined by CCK-8. ** *p* < 0.01, *** *p* < 0.001.

After the encapsulation feasibility of the scheme was confirmed by TEM and CLSM, the bacterial growth curve in the mixed medium was used to determine whether the influence of each polyphenol material on bacterial growth was obvious. As shown in Figure 4A, the three polyphenol materials had different effects on the growth curve of *Lactobacillus plantarum*. Among them, the growth curve of bacteria in the TP group was the closest to that in the control group, while the growth of bacteria in the ACN and TA groups was significantly inhibited. TA inhibits the growth of Gram-positive and Gram-negative bacteria by directly binding to the peptidoglycan layer, destroying the integrity of the cell wall, or suppressing the activity of enzymes related to cell wall synthesis [40,41,42]. The antibacterial effect of ACN may be achieved by binding to lipopolysaccharides on the outer membrane of the bacteria or inhibiting the metabolism of the bacterial biofilm to clear the biofilm, thereby destroying the integrity of the outer membrane [43,44].

Then, the growth trend of bacteria in the carrier was observed to determine whether the coating had an effect on bacterial growth. As shown in Figure 4B, the growth trends of the two groups with TP and ACN as raw materials were similar after encapsulation, and the growth of bacteria was inhibited to a certain extent after encapsulation by TA. Compared with the results in Figure 4A, the Fe(III)-TA group showed a more obvious inhibitory effect after encapsulation, and the initiation time of the inhibition was significantly advanced, but the inhibitory effect was weakened. The mechanism of tannic acid inhibiting bacterial growth is not only inhibiting cell wall synthesis and destroying its integrity but also coupling with iron in the environment, thereby reducing the utilization of iron in the environment by cells and thus inhibiting their growth [45]. When Fe (III) forms an octahedral complex with polyphenols and does not adhere to the surface of the bacteria, it will cause certain toxicity to them and inhibit their growth. After encapsulation, in the added ferric chloride solution, most of the groups in TA are occupied by Fe (III), reducing the adsorption of Fe (III) by the culture medium and weakening the inhibitory effect on the growth of bacteria.

At the same time, as shown in the results of the cck-8 experiment (Figure 4), the cell proliferation activity in this experiment was generally consistent with the growth trend reflected in the cell growth curve in the first 4 h, outlined in Figure 4B. The bacterial activity of the Fe(III)-TP group was close to that of the Fe(III)-TA group at 1 h, and the change was obvious at 3–4 h, and the absorbance of the Fe(III)-TP group was close to that of the control group. After 4 h of culture, the medium in the 96-well plate showed an obvious orange-yellow color, and the cell proliferation in the Fe(III)-ACN group was more obvious, but the gap was larger than that in the blank group, while the growth activity in the 4 h before tannic acid was not significantly changed and was maintained at a low level. Yang Xinyuan prepared ECN@TA-Ca^2+^@Mucin carrier [30], and its colony count was higher than that of the control group, and the difference was significant after digestion. Compared with the effect of the Fe(III)-TA carrier in this experiment, the inhibitory capacity of the MPN system formed by tannic acid and calcium ion is weaker than that of the MPN system formed by tannic acid and iron ion. This indicated that tannic acid as the encapsulation material had a relatively significant inhibitory effect on the activity of *Lactobacillus plantarum* YJ7. Similarly, proanthocyanidins had a certain effect on the cell membrane structure, so the activity was decreased compared with the control group.

### 3.5. In Vitro Storage Experiments

**Figure 5:**
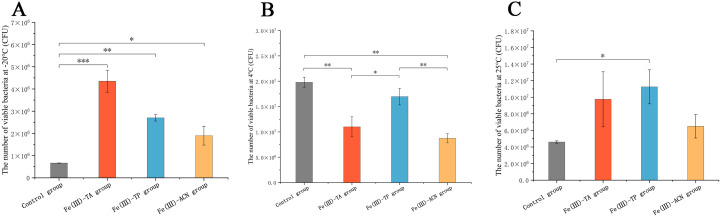
In vitro storage experiments of single-cell encapsulation carriers. (**A**) The number of remaining viable bacteria in each group after 7 days of storage at −20 °C. (**B**) The number of remaining viable bacteria in each group after 7 days of storage at 4 °C. (**C**) The number of remaining viable bacteria in each group after 7 days of storage at 25 °C. * *p* < 0.05, ** *p* < 0.01, *** *p* < 0.001.

In order to study the stability of the single-cell carrier after encapsulation, the carrier was stored in vitro for 7 days at different temperatures such as −20 °C, 4 °C, and 25 °C. The plate colony count results are shown in Figure 5. It can be seen that at 4 °C, the number of viable bacteria in the polyphenol group was lower than that in the control group, but there was no significant difference between the Fe(III)-TP group and control group. At 25 °C and −20 °C, the number of viable bacteria in each group was greater than that in the control group, and the storage effect was ideal. The Fe(III)-TA group has a remarkable storage effect at −20 °C, indicating that the MPN system of Fe(III)-TA has a stronger tolerance to low temperatures. This might be because low temperatures slow down the metabolic rate of microorganisms, reduce the death of strains, and thus maintain their activity. On the other hand, it might be that the thermal motion of molecules decreases at low temperatures, which slows down the migration of molecules within the MPN system and hinders several diffusion-controlled reactions. A similar study has found that Fe(III)-TA MPN could improve the hydration layer’s preservation and shield bacteria from damage [46]. Zhao et al. also found that the activity of microcapsules was not affected at −20 °C [33].

### 3.6. The Survival Ability for Gastric Assaults

**Figure 6:**
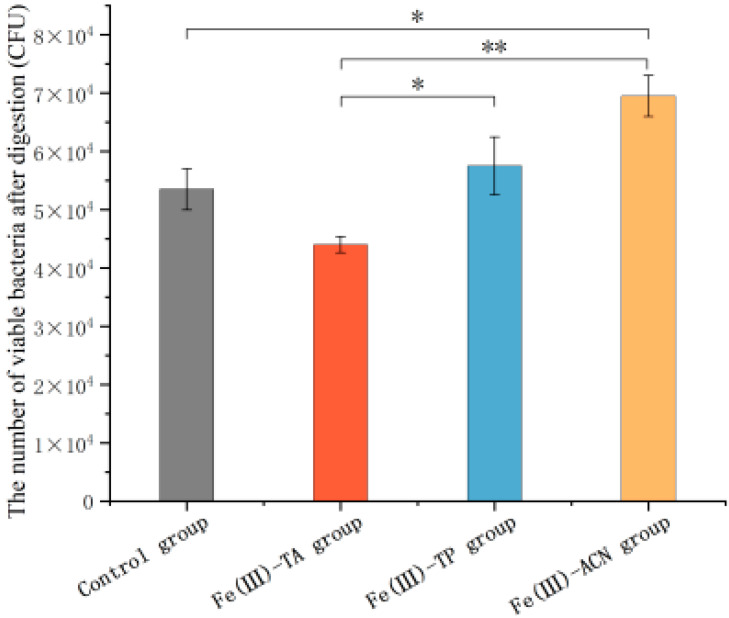
Viable bacteria count of single-cell delivery carriers in vitro test with simulated gastric fluid. * *p* < 0.05, ** *p* < 0.01.

The stomach is one of the most important parts of human digestion, which can secrete highly acidic gastric acid. Most probiotics will be inactivated in this extreme environment, which is one of the biggest difficulties for the human use of probiotics [47]. In this study, single-cell delivery carrier samples were placed in artificial gastric juice to simulate the process of probiotics in the stomach environment after administration. As shown in Figure 6, the viable bacterial count in the control group was 5.35 × 10^4^ ± 3.54 × 10^3^ CFU, while that in the Fe(III)-TA, Fe(III)-TP, and Fe(III)-ACN groups was 4.40 × 10^4^ ± 1.41 × 10^3^ CFU, 5.75 × 10^4^ ± 4.95 × 10^3^ CFU, and 6.95 × 10^4^ ± 3.54 × 10^3^ CFU, respectively. A statistically significant enhancement in cell survival was observed in the Fe(III)-ACN-coated group compared with the control group (*p* < 0.05). These results further indicated that the tolerance of *Lactobacillus plantarum* to the human digestive tract environment was significantly improved after the anthocyanin-based MPN system was encapsulated [48].

## 4. Conclusions

Interestingly, TA and ACN initially inhibited bacterial growth, but post-encapsulation, ACN’s inhibitory effect vanished, whereas TA’s effect diminished. CCK-8 assays confirmed that Fe(III)-TA significantly suppressed bacterial activity, followed by Fe(III)-ACN. These outcomes may be attributed to TA’s membrane-disrupting properties, which compromise bacterial integrity and enzymatic activity. In vitro simulations demonstrated that tea polyphenol-based MPNs preserved the highest viable bacterial counts at 25 °C. Notably, anthocyanin-based MPNs exhibited optimal encapsulation efficiency for *L. plantarum YJ7*. This is not only because the inhibitory effect of anthocyanin-based MPNs on *L. plantarum YJ7* is weakened or even disappears and the number of viable bacteria at −20 °C and 25 °C is better than that of the naked bacteria group but because, more importantly, anthocyanin-based MPNs show a better number of viable bacteria than Fe(III)-TA and Fe(III)-ACN during artificial gastric juice simulated digestion. This study introduces a novel strategy for probiotic encapsulation, selecting tea polyphenol-iron MPNs as optimal materials. However, this study has certain limitations, such as the need for further confirmation of the fixation effect of iron ions in MPNs. Moreover, future research should prioritize evaluating the colonization ability and functional performance of these vectors in the gastrointestinal tract.

## Data Availability

The original contributions presented in this study are included in the article. Further inquiries can be directed to the corresponding author.
